# Pulmonary inflammatory response and immunomodulation to multiple trauma and hemorrhagic shock in pigs

**DOI:** 10.1371/journal.pone.0278766

**Published:** 2022-12-07

**Authors:** Marc-Alexander Oestreich, Kerstin Seidel, Wilhelm Bertrams, Hans-Helge Müller, Martin Sassen, Thorsten Steinfeldt, Hinnerk Wulf, Bernd Schmeck

**Affiliations:** 1 Institute for Lung Research, Universities of Giessen and Marburg Lung Center, German Center for Lung Research (DZL), Philipps University Marburg, Marburg, Germany; 2 Vascular Biology Section, Evans Department of Medicine, Whitaker Cardiovascular Institute, Boston University School of Medicine, Boston, MA, United States of America; 3 Institute for Medical Bioinformatics and Biostatistics, Philipps-Universität Marburg, Marburg, Germany; 4 Department of Anesthesia and Intensive Care Medicine, University Medical Center Gießen and Marburg, Philipps University Marburg, Marburg, Germany; 5 Center for Emergency Medicine, University Medical Center Gießen and Marburg, Philipps University Marburg, Marburg, Germany; 6 BG Unfallklinik Frankfurt am Main gGmbH, Department for Anesthesia, Intensive Care Medicine and Pain Therapy, Frankfurt am Main, Germany; 7 Department of Pulmonary and Critical Care Medicine, University Medical Center Giessen and Marburg, German Center for Lung Research (DZL), Philipps University Marburg, Marburg, Germany; 8 Center for Synthetic Microbiology (SYNMIKRO), Philipps-University of Marburg, Marburg, Germany; 9 German Center for Infection Research (DZIF), Partner Site Giessen-Marburg-Langen, Marburg, Germany; University of Western Ontario, CANADA

## Abstract

**Background:**

Patients suffering from severe trauma experience substantial immunological stress. Lung injury is a known risk factor for the development of posttraumatic complications, but information on the long-term course of the pulmonary inflammatory response and treatment with mild hypothermia are scarce.

**Aim:**

To investigate the pulmonary inflammatory response to multiple trauma and hemorrhagic shock in a porcine model of combined trauma and to assess the immunomodulatory properties of mild hypothermia.

**Methods:**

Following induction of trauma (blunt chest trauma, liver laceration, tibia fracture), two degrees of hemorrhagic shock (45 and 50%) over 90 (n = 30) and 120 min. (n = 20) were induced. Animals were randomized to hypothermia (33°C) or normothermia (38°C). We evaluated bronchoalveolar lavage (BAL) fluid and tissue levels of cytokines and investigated changes in microRNA- and gene-expression as well as tissue apoptosis.

**Results:**

We observed a significant induction of Interleukin (IL) 1β, IL-6, IL-8, and Cyclooxygenase-2 mRNA in lung tissue. Likewise, an increased IL-6 protein concentration could be detected in BAL-fluid, with a slight decrease of IL-6 protein in animals treated with hypothermia. Lower IL-10 protein levels in normothermia and higher IL-10 protein concentrations in hypothermia accompanied this trend. Tissue apoptosis increased after trauma. However, intervention with hypothermia did not result in a meaningful reduction of pro-inflammatory biomarkers or tissue apoptosis.

**Conclusion:**

We observed signs of a time-dependent pulmonary inflammation and apoptosis at the site of severe trauma, and to a lower extent in the trauma-distant lung. Intervention with mild hypothermia had no considerable effect during 48 hours following trauma.

## Introduction

Injuries and traumata affect all age groups, but especially the young, and remain among the top ten causes of death [1]. These approximately 5.8 million deaths per year account for 10% of global deaths and constitute a public health burden [2, 3]. In Germany, 14% of trauma patients meet the definition of a polytrauma indicating a life-threatening situation due to a combination of multiple injuries and a physiological response [4–6]. Often, these injuries are accompanied by ischemia due to hemorrhagic shock, fractures and soft tissue injury as well as organ contusion. Therefore, polytrauma patients bear a high risk of developing post-traumatic complications (e.g. sepsis or organ failure) which further complicate treatment [7].

Numerous studies emphasize the central role of the lung in the posttraumatic development of organ dysfunction and failure, defined as multiple organ dysfunction syndrome (MODS) or multiple organ failure (MOF) [8]. This is particularly of interest as almost half of severe trauma patients suffer direct trauma to the chest, often accompanied by lung contusion, which can aggravate respiratory complications and their progression to early deterioration of lung function or even acute respiratory distress syndrome (ARDS) [5, 8]. In addition to direct injury, the lung is also of relevance in patients without lung contusion, as trauma-distant or remote organ damage is associated with the posttraumatic inflammatory response [9].

Nowadays, as victims of multiple trauma and hemorrhagic shock frequently survive the initial insult [5], the mechanisms of this posttraumatic, often exuberant immune response and the subsequent deterioration of lung function have come into research focus [10]. But despite promising results for the early posttraumatic phase, few studies have examined effects of combined trauma and shock in a clinically relevant model after more than 24 hours [11, 12]. While previous studies focused on observation periods of up to 24 hours with intubated animals [13–15], other protocols had animals extubated after 24 hours followed by several hours or even days of awake observation [16, 17]. However, this does not reflect the clinical situation of most polytrauma patients, who are intubated and treated in the ICU for several days [5], and studies with observational periods exceeding 36 or 48 hours with intubated animals suffering combined shock and trauma remain scarce. The longitudinal kinetics of markers of the complex post-traumatic immune response, such as concentrations of inflammatory cytokines, and the expression of key transcription factors and their regulators (e.g. microRNAs) can only be investigated in studies with clinically relevant observation periods [11]. Given that impaired regulation of the immune response after trauma may also extend to remote organ damage [9], regulators of the immune response are of particular interest. MicroRNAs 146a and 155 form a regulatory circuit of NF-κB, which regulates NF-κB activity and thus the intensity and duration of the inflammatory response (e.g. by influencing TLR3/4 signaling pathways and regulating IRAK1 and TRAF6) [18–20].

In addition, potential immunomodulatory effects of therapeutic hypothermia (a core body temperature below 35°C) during the first days of treatment are not yet fully understood. While experimental studies and trauma models focus on quantitative outcomes during the early posttraumatic phase, clinical studies report long-term patient-oriented outcomes (e.g. adverse effects or mortality). This discrepancy, as well as the lack of studies with long observation periods and a clear protective effect, has resulted in therapeutic hypothermia currently not being recommended by WHO [21], NICE [22], or AWMF [23] guidelines.

Therefore, in this study we aimed to investigate the pulmonary inflammatory response to multiple trauma and hemorrhagic shock in a clinically relevant porcine trauma model over 48 hours. In addition, we examined whether an intervention with mild therapeutic hypothermia (33° C) would attenuate the respiratory inflammatory response and tissue damage.

## Materials and methods

In accordance with the principle of restriction, refinement and reduction for animal studies, the data from experimental animals presented in this study has been collected as part of a multi-center study on severe multiple trauma from which different results have been published by our collaborators [24–29]. For this investigation, we used data of 60 male pigs (Sus scrofa domesticus) with a mean body weight of 35±5 kg and an age of 3 to 6 months.

### Ethics

We strictly followed the experimental protocol as approved by local ethics and animal care committees of the District Government of Giessen, Germany (Protocol Number MR 20/17, Ref. 22/2013) which was developed based on the Helsinki convention for the use and care of animals and reported in consent with the ARRIVE guidelines. Complications were addressed according to current standards of emergency medicine and trauma surgery, as recommended by the European Resuscitation Council as well as in Advanced Trauma Life Support (ATLS) protocols. Animals received typical emergency medications (i.e., epinephrine, noradrenaline, amiodarone, midazolame, and atropine) in life-threatening events (i.e. seizure, tension pneumothorax, ventricular fibrillation, cardiac arrest). Resuscitation was terminated after 45 min. without achieving return of spontaneous circulation.

### Experimental groups

**Fig 1:**
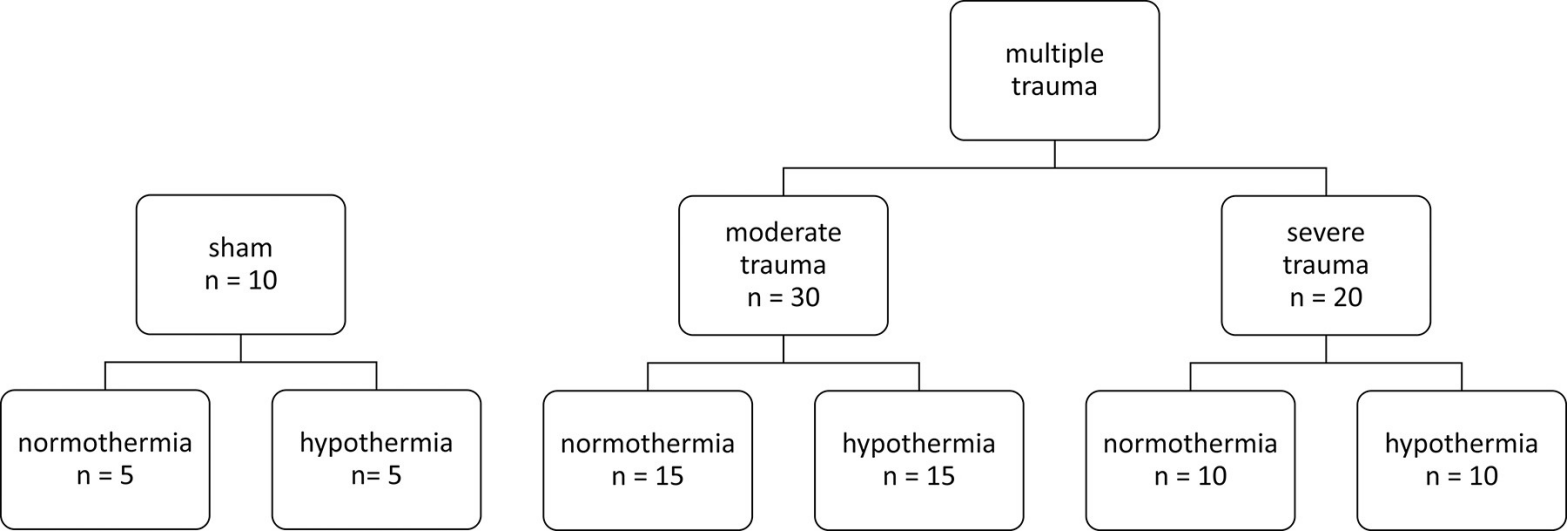
**Study groups.** Sixty animals were randomly assigned to six study groups. Sham represents the placebo surgery group. Multiple trauma included lung contusion, liver laceration, tibial fracture, and hemorrhagic shock (moderate or severe). Intervention with mild hypothermia (33° C) for 12 hours and subsequent rewarming over 10 hours. Animals in the normothermia group were kept at 38° C core temperature.

We analyzed data from six different groups from a standardized polytrauma model as previously described by Eschbach et al. [25]. Ten non-traumatized pigs served as controls: sham-NT (NT = Normothermia; n = 5) and sham-HT (HT = Hypothermia; n = 5; Fig 1). Standardized moderate trauma with less blood loss included lung contusion, liver laceration, lower leg fracture and a hemorrhage with a blood volume loss of 45% and a mean arterial pressure (MAP) < 30 mmHg over a shock time of 90 min. (moderate trauma-NT (n = 15) and moderate trauma-HT (n = 15)). These conditions were escalated in a severe trauma group that underwent the same standardized procedure but suffered a blood volume loss of 50% with a MAP <25 mmHg over 120 min. (severe trauma-NT (n = 10) and severe trauma-HT (n = 10)). Animals were randomly assigned to either intervention or control groups.

### Experimental protocol

Anesthesia, monitoring, trauma induction, observation and sample collection were conducted by our collaborators as previously described [25]. After a fasting period of 12 h, the pigs were pre-medicated with diazepam (1 mg/kg), ketamine (20 mg/kg) and atropine (0.5 mg) i.m., brought into prone position and pre-oxygenated (10 L O_2_/min). Following cannulation of the ear vein, anesthesia was induced and held up continuously using sufentanil (0.8 μg/kg/h) and disoprivane (3–4 mg/kg/h) with additional midazolam 2% if indicated by shivering. The animals were intubated (7.5 Charrière tube) and underwent continuous pressure-controlled ventilation with a tidal volume of 6–8 mL/kg (Draeger, Evita, Danvers, MA, USA), positive end-expiratory pressure of 5 cm H_2_O, FiO_2_ of 0.3, and inspiration-to-expiration ratio of 1:2. The respiratory rate was adjusted to maintain an end-tidal CO_2_ between 45 to 55 mmHg. Ventilatory parameters were evaluated every two hours using blood gas analysis (BGA, ABL700, Radiometer, Copenhagen). Continuous infusion of a balanced saline solution (Ringer´s acetate) with a rate of 2 mL/kg/h was held up during the entire study time with a bolus of 10 mL/kg in case of hypovolemia. Antibiotic prophylaxis with cefuroxime (80 mg/kg) was administered once. Vital signs were monitored by electrocardiogram-synchronized pulse oximetry, ECG recording and naso-oesophageal temperature probe. Subsequently, aseptic insertion of an arterial PiCCO system (pulse contour cardiac output) in the left femoral artery, a central venous line via the right jugular vein (3 lumen HD, Arrow, PA, USA), a suprapubic urine catheter, and a two-lumen hemodialysis line in the left femoral vein (2 lumen HD, 14 Charrière, 15 cm, Arrow, PA, USA) and tracheotomy were undertaken.

For standardized induction of trauma, the right hind leg was placed in a drop-weight device and a tibia fracture including soft-tissue damage was induced by a 20 kg plumb-cuboid, which was dropped from a height of 100 cm. Blunt trauma of the right thorax was performed by launching a captive bolt stunner (Dynamite Nobel GmbH, Troisdorf, Germany) with direct contact to a 10 x 10 cm panel (upper layer of lead, lower layer of steel). Subsequently, laparotomy was performed including two lacerations of the liver (caudal lobe). After 30 sec., bleeding was stopped using a tamponade. Simultaneously, systemic hemorrhage was induced by drawing blood from the femoral vein until reaching a MAP of 30±5 mmHg in moderate trauma groups and a MAP of 25±5 mmHg in severe trauma groups. Hemorrhagic shock was held up for either 90 (moderate) or 120 min. (severe trauma). Following trauma, reperfusion with warmed Ringer´s acetate solution over one hour (4 times the hemorrhagic volume) took place. Sham animals were handled accordingly but received neither injuries nor hemorrhage nor did they receive reperfusion.

### Intervention with hypothermia

**Fig 2:**
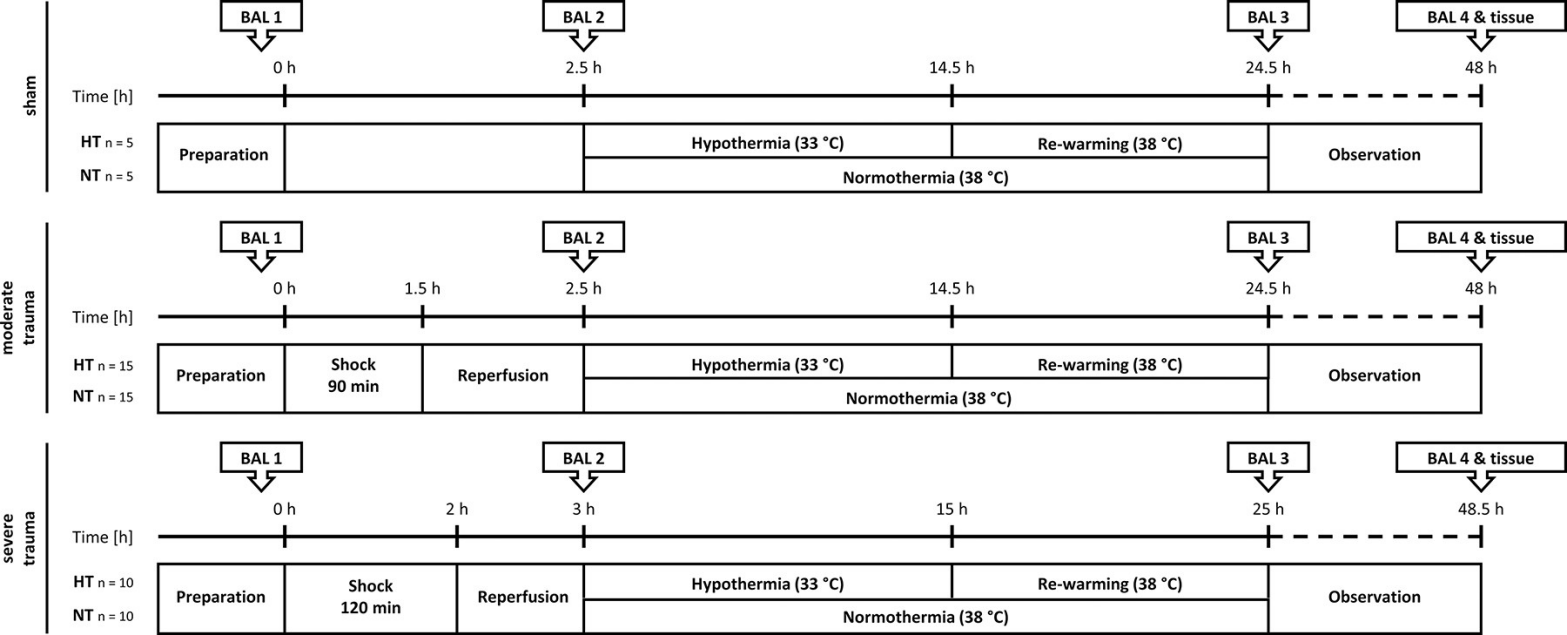
Experimental groups. Animals underwent preparation and standardized trauma induction (including blunt chest trauma, liver laceration, and tibial fracture). Hemorrhagic shock was induced by blood volume removal until reaching a mean arterial pressure of 30±5 mmHg over 90 min. in moderate and 25±5 mmHg over 120 min. in severe trauma groups. Following resuscitation and reperfusion with warmed Ringer´s acetate solution, animals were randomized to either intervention with mild hypothermia and subsequent rewarming or normothermic observation. Sham animals were not injured. In the hypothermia groups, the body temperature was reduced to 33° C. After rewarming at 0.5° C per hour over 10 hours, animals were observed for additional 24 hours. Bronchoalveolar lavage (BAL) fluid was sampled before trauma induction (BAL1), after reperfusion (BAL2), after rewarming (BAL3), and at the end of the observational period (BAL4), respectively. Lung tissue samples were taken at the end of the observational period.

Controlled, mild hypothermia was induced using the Arctic Sun 5000 temperature management system (Bard Medical, Medivance, Louisville, KY, USA) by placing two hydrogel pads on the animal’s flanks. The animals were cooled for a period of 12 hours, reaching a core body-temperature of 33°C within 3 hours, followed by a rewarming period (0.5°C/h) over 10 hours (Fig 2). After rewarming, the animals were observed for 24 hours until induction of euthanasia by administering additional sufentanil (100 μg), disoprivane (200 μg) and pancuronium (4 mg) followed by potassium chloride (60–100 mL) until the onset of cardiac arrest.

### Sample collection and preparation

Bronchoalveolar lavage (BAL) fluid samples were collected immediately before induction of trauma (BAL1), after shock and reperfusion (BAL2), following re-warming (BAL3) and after another 24 hours of observation (BAL4) by positioning the pigs on the right side and performing lavage to the right lung using a kinked suction catheter. Using a 50 mL syringe, 30 mL 0.9% NaCl-solution were instilled and immediately re-aspirated. Samples were then cooled, prepared for storage, centrifuged (4°C, 5 min, 4000 rpm), and stored at -80° C. Immediately after euthanasia, lung tissue samples were taken from three defined locations of the lungs (contusion-site, ipsilateral upper lobe, contralateral upper lobe), shock frosted in liquid nitrogen, and stored at -80° C.

Concentration of BAL-fluid samples was performed using Amicon^®^ Ultra-4 centrifugal filter devices (Merck, Darmstadt, Germany) with a cut-off of three kDa. Correction of cytokine-concentration was based on total protein concentration (determined by Pierce^©^ BCA protein assay kit, Thermo Fisher Scientific, Rockford, IL, USA) and sample volume before and after concentration and is given volume-independent in [pg cytokine / μg total protein]. Baseline values for IL-10 of the sham group were below the lower detection limit of 31.2 pg/ml and set to 15.6 pg/ml (^1^/_2_ of the lower detection limit).

Lung tissue was shock frozen in liquid nitrogen, mortared, partitioned and weighed. For total protein extraction, we used T-PER^©^ tissue protein extraction reagent (Thermo Fisher Scientific, Rockford, IL, USA) in a 1:5 ratio (^w^/_v_). Samples were then homogenized, vortexed, incubated, sonicated (5 cycles of 30 seconds each at 4°C with 5 watts (Biorupter Sonicator, Diagenode SA, Seraing, Belgium)), and centrifuged. After determining the protein content, the supernatant was stored at -80° C.

### RNA preparation and real-time qPCR

Total RNA isolation was performed by phenol-chloroform extraction with Tri Reagent (Merck, Darmstadt, Germany), quantified by Nanodrop^®^ (Thermo Fisher Scientific, Rockford, IL, USA), assessed for quality using total RNA Nano Chip (Agilent 2100 Bioanalyzer, Agilent Technologies, Santa Clara, CA, USA) and reverse transcribed with the High Capacity cDNA Reverse Transcription kit or the microRNA reverse transcription kit (both Thermo Fisher Scientific, Rockford, IL, USA). Quantitative real-time PCR was performed on a ViiA7^©^ (Thermo Fisher Scientific, Rockford, IL, USA) using Fast SYBR Green Master Mix (Thermo Fisher Scientific, Rockford, IL, USA) and primer pairs (Metabion International, Planegg/Steinkirchen, Germany) for Interleukin-1, Interleukin-6, Interleukin-8, Surfactant protein-C, IKBA, Thioredoxin, and Cyclooxygenase-2 (S1 Table in S1 File). β-actin was used as an endogenous control for mRNA analyses. For analyses of microRNAs 146a and 155, we identified ssc-microRNA-17-5p as a stable endogenous control. MicroRNA primer sequences were previously described by Mentzel et al. [30]. Samples were measured in technical triplicates. Data were processed with the ViiA7^®^ software v 1.2.4 (Life Technologies) and analyzed using the ΔΔ C_T_ method [31].

### ELISA

Cytokines (IL-6, IL-10) in BAL-fluid and supernatant of lung tissue were analyzed with commercial ELISA kits (DuoSet^©^ ELISA, R&D Systems, Abingdon, UK) following identification in a Bio-Plex Magpix (Luminex Corp., Austin, USA) according to manufacturer’s instructions.

### Western blot

Protein concentrations were determined using the Pierce BCA protein assay kit (Thermo Fisher Scientific, Rockford, IL, USA). Equal amounts of protein (60 μg) were separated in 15% Tris-HCl SDS-polyacrylamide gels for semi-dry blotting of caspase-3 with 0.003 mA/cm^2^ on PVDF membranes. Prior to incubation with antibodies, the blots were cut horizontally along 38 kDa to enable simultaneous analysis of ß-actin (40 kDa), pro-caspase-3 (32 kDa) and cleaved caspase-3 subunits (11, 17, 20 kDa), washed and blocked (3% bovine serum albumin in TBS-T_20_). The chemo-luminescent signals were captured using a Gel-x imager system (Intas Science Imaging Instruments, Göttingen, Germany), quantified by densitometry (ImageJ 1.52j, National Institutes of Health, USA) and normalized to ß-actin. Untreated and cytochrome c-treated Jurkat control cell extracts (#9663, Cell Signaling Technology, Frankfurt, Germany) served as controls. Primary monoclonal antibodies for caspase-3 (E8, #7272, 1:1000) and ß-actin (#1616, 1:1000) were obtained from Santa Cruz Biotechnology (Heidelberg, Germany).

### Statistical analysis

The primary outcome of our study was the characterization of the pulmonary inflammatory response (quantified by BAL fluid levels of cytokines IL-6 and IL-10, changes in gene-expression of IL-1, IL-6, IL-8, SP-C, IKBA, TXN, and COX-2 and miRNA-146 and -155, as well as tissue caspase-3 levels) to multiple trauma and hemorrhagic shock under normothermic conditions. Secondary outcomes were i) the characterization of immunomodulatory effects of mild hypothermia on cytokine-levels, gene and microRNA expression, and lung tissue apoptosis compared to normothermia and ii) the characterization of a systemic activation of inflammatory response regulators (microRNA expression) and markers of lung tissue apoptosis (caspase-3) in the non-injured left lung and the contused right lung.

Distribution of data has been investigated by histograms. Logarithmic transformation was performed for all statistical analyses to approximate a Gaussian normal distribution. We applied the closed testing principle to reduce the number of tests [32]. A detailed description of all analyses is provided in the online supplement. In brief, the trauma groups were compared by a global Kruskal-Wallis test on adjusted BAL fluid cytokine-, gene expression-, microRNA expression-, and tissue apoptosis-data. Significant differences (p≤0.05) were further differentiated by a Wilcoxon-Mann-Whitney test. Additionally, differences between the treatment groups were analysed by a Wilcoxon-Mann-Whitney test and differences between locations were analysed by a Wilcoxon matched-pairs signed rank test.

Data processing and analyses were performed in Stata 16 (StataCorp LLC, College Station, USA) and GraphPad Prism 8.0 (GraphPad, La Jolla, USA). Statistical significance was assumed at p≤0.05. Data are presented as mean+SD if not indicated otherwise.

## Results

During the study, four experimental animals died within the first 12 hours of the study protocol and were excluded from this analysis (two in Trauma 1 NT, two in Trauma 2 HT). Physiological parameters, clinical characteristics, and mortality have previously been described [24, 26–29].

### Pulmonary inflammatory response to multiple trauma and hemorrhagic shock

**Fig 3:**
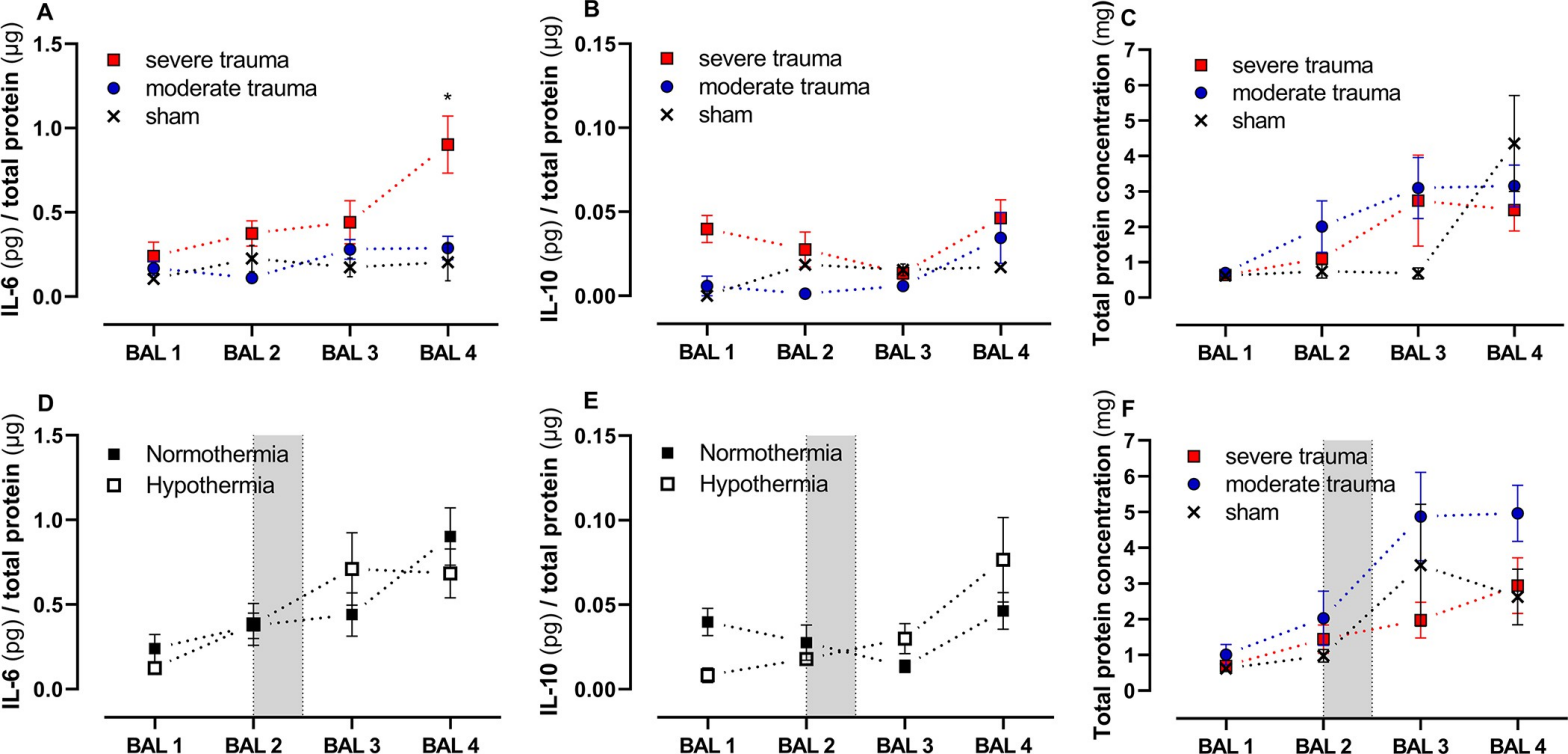
Time dependent activation of the pulmonary inflammatory response. Bronchoalveolar lavage fluid was sampled at four time points (before (BAL1) and after trauma (BAL2), after 24 (BAL3) and 48 hours (BAL4)), concentrated, and analyzed by ELISA. The mean values±SEM are shown. Kinetics of IL-6 (A) and IL-10 (B) concentrations [pg cytokine/μg total protein] for sham, moderate trauma, and severe trauma and total protein (C; [mg]) under normothermic conditions over the course of the study period are shown. IL-6 concentration in BAL fluid increased significantly in the severe trauma group (p = 0.0373) over the course of 48 hours. Panel (D) and (E) depict kinetics of IL-6 and IL-10 concentrations of the severe trauma group comparing normo- and hypothermia treatment. The shaded area between BAL 2 and BAL 3 indicates the intervention with mild, controlled hypothermia over 12 hours. Figure (F) shows mean±SEM total protein concentrations [mg] for sham, moderate trauma, and severe trauma with hypothermia treatment. Statistical analysis: Closed-testing principle of adjusted BAL4 values: Kruskal-Wallis (p≤0.05) and Wilcoxon-Mann-Whitney (p≤0.05) test. Detailed information including sample counts are provided in S2 and S3 Tables in *S1 File*.

Following severe trauma, the BAL fluid concentration of IL-6 increased over the course of 48 hours (Fig 3A and 3C). There were significantly higher IL-6 protein levels in the severe trauma group when compared to moderate trauma at the end of the study period (48 hours: 3.1-fold increase; p = 0.016, S2 Table in S1 File). Likewise, the IL-10 concentration was elevated in severe compared to moderate trauma over the whole study period, but this trend did not reach statistical significance (p = 0.194; Fig 3B and 3C).

**Fig 4:**
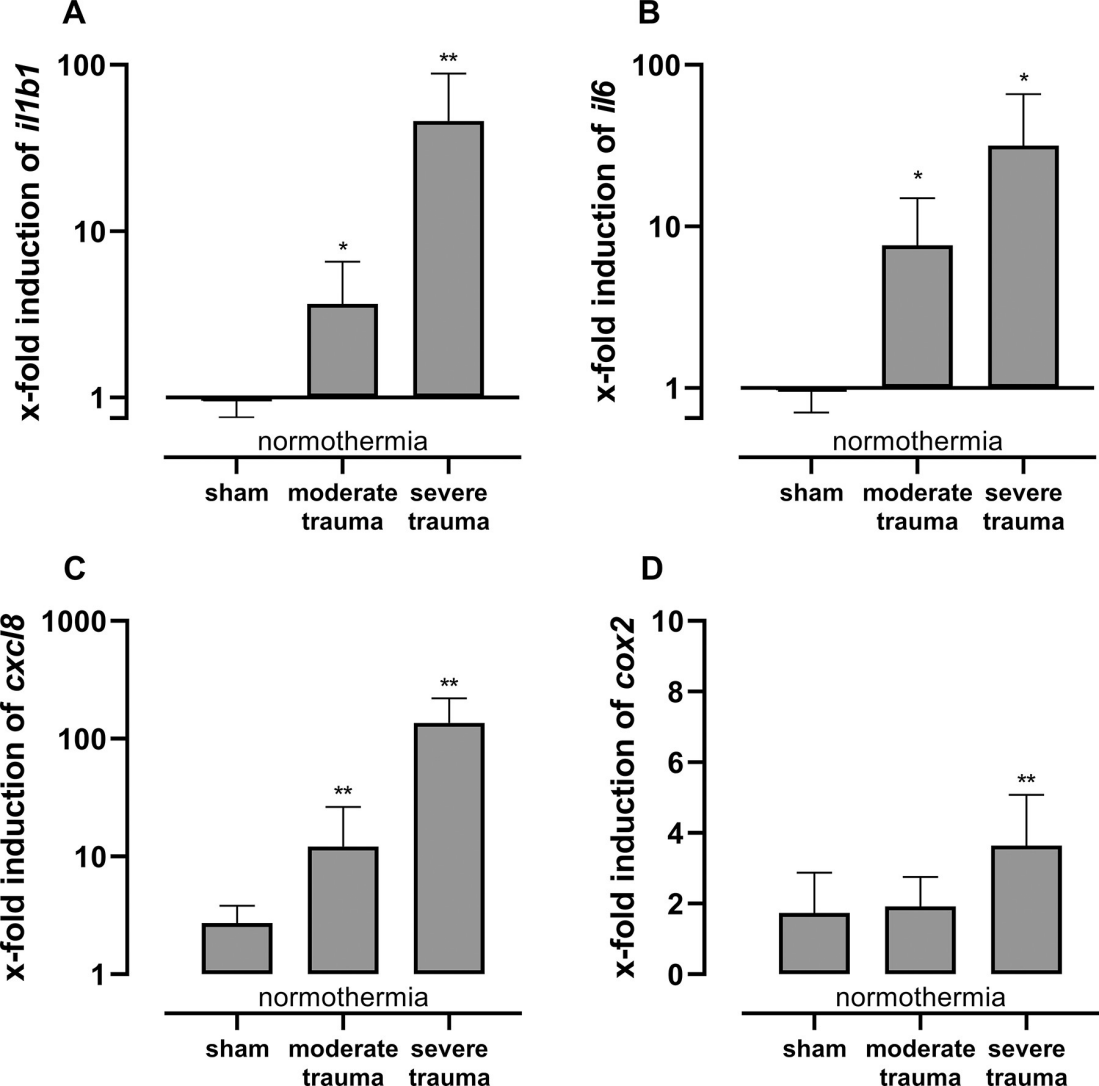
Multiple trauma and hemorrhagic shock induce a transcriptional immune response. Gene expression [x-fold] of pro-inflammatory biomarkers IL-1β (A), IL-6 (B), IL-8 (C), and Cyclooxygenase-2 (D). Lung tissue homogenates from the right upper lobe (ipsilateral control) were analyzed by RT-qPCR, logarithmic data were normalized to β-actin. Mean (SD) values on log10 (A, B, C) and linear scale (D) are shown. Statistical analysis: Closed-testing principle: Kruskal-Wallis (p≤0.05) and Wilcoxon-Mann-Whitney (p≤0.05) test: *p≤0.05, **p≤0.01. Detailed information including sample counts, additional genes, and other locations (contusion site, contralateral control) are provided in S4 and S5 Tables in *S1 File*.

Trauma and hemorrhagic shock induced a transcriptional immune response in the contused lung (site of lung contusion and ipsilateral control). The gene expression of pro-inflammatory biomarkers (IL-1β, -6 and -8) was significantly increased in both the moderate and severe trauma groups when compared to sham controls (ipsilateral control; Fig 4A–4C, S4 Table in S1 File), Cyclooxygenase-2 expression was increased in the severe trauma group (Fig 4D, S4 Table in S1 File). Anti-inflammatory IκBα, Thioredoxin, and Surfactant-associated protein C showed no relevant differences (S4 Table in S1 File).

Apoptosis, quantified by caspase-3 cleavage, was significantly increased in injured lungs compared to lungs from the sham group (p = 0.001; S2B Fig and S8 Table in S1 File) but not at the contusion site or the contralateral control.

### Trauma-distant response to multiple trauma and hemorrhagic shock

**Fig 5:**
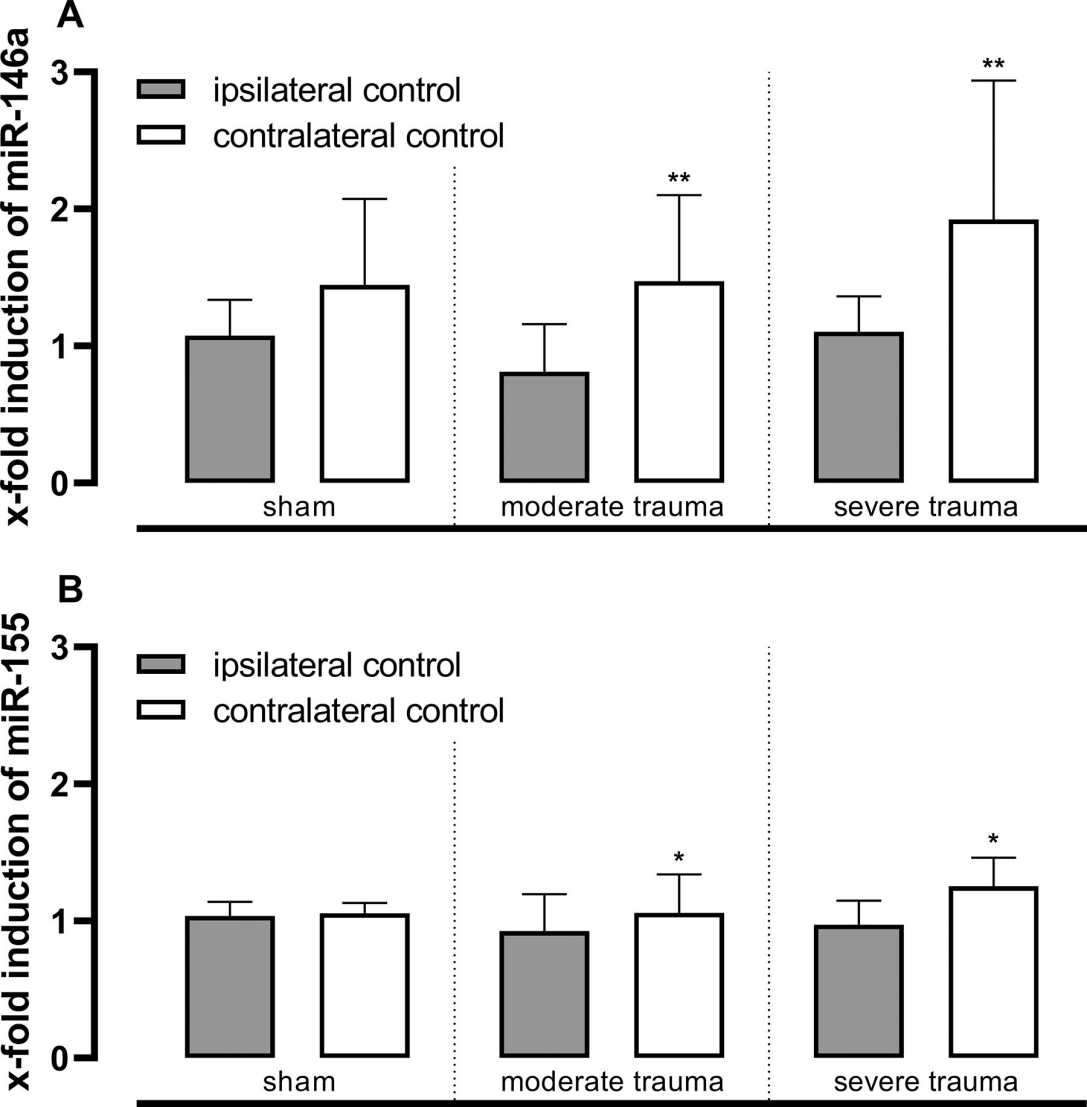
Trauma-distant activation of inflammatory response regulators in the non-injured left lung. Expression of microRNA-146a and microRNA-155 [x-fold]. Lung tissue homogenates from the right upper lobe (ipsilateral control) and the non-injured left upper lobe (contralateral control) were analyzed by RT-qPCR, logarithmic data were normalized to microRNA-17. Mean (SD) values on linear scale are shown. Statistical analysis: Mann-Whitney test: *p≤0.05, **p≤0.01. Detailed information including sample counts are provided in S6B Table in *S1 File*.

After trauma, lung tissue samples from the left upper lobe (trauma-distant control) showed increased expression of miRNA-146a (moderate trauma: 0.6-fold, p = 0.002; severe trauma: 0.8-fold, p = 0.008; Fig 5A) and miRNA-155 (moderate trauma: 0.2-fold, p = 0.027; severe trauma: 0.3-fold, p = 0.016; Fig 5B), when compared to the right upper lobe (trauma-near control). There was no considerable effect on tissue apoptosis (caspase-3 western blot) or transcriptional immune response (IL-1β, -6 and -8, COX-2, IκBα, TXN, and SP-C) between the injured and sham lungs (S8 Table in S1 File).

### Immunomodulatory effects of mild hypothermia

We observed a trend towards higher IL-6 protein concentrations in BAL fluid following severe trauma and normothermic treatment and a peak with subsequently declining levels following intervention with mild hypothermia (Fig 3D and 3F). Lower IL-10 protein levels in normothermia and higher concentrations in animals receiving hypothermia treatment accompanied this trend (Fig 3E and 3F). Besides these trends in BAL fluid cytokine kinetics, the intervention with mild hypothermia did not result in statistically significant effects on mRNA expression (S5 Table in S1 File), the expression of miRNA-146a or miRNA-155 (S7 Table in S1 File), nor activation of caspase-3 as a marker of lung tissue apoptosis (S9 Table in S1 File).

## Discussion

The pulmonary inflammatory response to severe multiple trauma has crucial impact on the onset of secondary organ impairment and failure [33, 34]. While most studies focus on changes within the first hours after trauma [11], we aimed to investigate the pulmonary inflammatory response (including the effects of induced mild hypothermia) in a clinically relevant porcine model with multiple trauma and moderate (45% blood loss over 90 min) or severe (50% blood loss over 120 min) hemorrhagic shock over 48 hours. Here, we complement findings of our collaborators, whose data demonstrated a time-dependent activation of the inflammatory response to multiple trauma with significant changes in cytokine and alarmin levels, both local (in fracture hematoma and brain tissue) and systemic (in blood serum) [24, 26–29].

Following severe multiple trauma, we observed an activation of the pulmonary inflammatory response (increased mRNA expression of IL-1β, IL-6, IL-8, and COX-2 as well as increased IL-6 protein in BAL fluid). Overall, therapeutic hypothermia had no significant effect on measures of the pulmonary immune response or tissue apoptosis.

Historically, most individuals suffering severe injury bled to death. With advances in immediate patient care and the control of hemorrhage and coagulopathy, trauma victims now frequently survive the initial insult but remain at risk of developing (multi-) organ failure and sepsis due to an over-exuberant immune response following the traumatic insult [10]. Previous studies highlighted the important role of pulmonary injuries, whether caused by direct lung injury or via remote organ injury, as a risk factor for the development of ARDS, but few examined the effects beyond 24 hours after combined trauma [11, 33].

In our study, multiple trauma and hemorrhagic shock induced an early pulmonary inflammatory response that aggravated over the course of the observational period. We found elevated levels of pro-inflammatory biomarkers in BAL fluid and lung tissue samples. This is in accordance with previously reported findings of significantly higher blood serum levels of IL-6 [26, 27] and High-Mobility Group Protein B-1 (HMGB-1) as well as decreasing concentrations of Heat Shock Protein 70 (HSP70) [28] after multiple trauma and in accordance with clinical observations [8].

We found increased mRNA expression of pro-inflammatory IL-1β, IL-6, IL-8, and COX-2 after induction of severe multiple trauma (Fig 4). Compared to the animals with moderate trauma, the combination of intensified hemorrhage (50% vs. 45%) and a prolonged shock time (120 instead of 90 minutes) resulted in a considerably stronger inflammatory response. These results are in line with findings of other studies with observational times above 24 hours that reported activation of both local and systemic inflammatory responses to trauma in porcine models [12, 35].

Signaling of the transcription factor NF-κB plays a central role in inflammation and cytokine upregulation [36]. Mechanistically, various stimuli (e.g. ligands of cytokine- and pattern recognition-receptors) activate the canonical pathway in which degradation of IκBα (via phosphorylation by the IκB kinase complex) activates NF-κB, which in turn regulates the inflammatory response [36]. In the present study, we observed a significant transcriptional induction of pro-inflammatory cytokines IL-1β, IL-6, IL-8, and COX-2 in lung tissue, while IκBα (porcine Nfkbia) showed no substantial changes.

In addition to the influence of transcription factors, gene expression can be regulated post-transcriptionally (e.g. translational repression or mRNA degradation) by mircroRNAs binding to 3′-untranslated regions [37, 38]. This is of particular importance given that microRNAs miR-146a and miR-155 were found to be involved in the regulation of NF-κB. Here, miR-155 (initially activated by NF-κB) acts as an immediate amplifier and positive regulator of NF-kB activity, while miR-146a functions as a time-delayed negative NF-κB regulator [20]. Together, both microRNAs form a regulatory circuit of NF-kB activity in inflammation [20]. In this study, we found that the expression of both, miR-146a and miR-155, was increased in trauma-distant lung tissue samples from both trauma groups, while IkBa revealed no substantial differences between trauma groups nor sampling locations, indicating that ongoing remote inflammation (as this lobe was not damaged during trauma induction or BAL sampling) may have led to an induction of initially miRNA-155 and thereafter miR-146a, which subsequently acted as a time-delayed negative regulator of the immune response. There was no substantial difference between trauma and sham control at the time of lung tissue sampling (48 hours after trauma). This study did not include other microRNAs with regulatory effects on inflammatory gene expression (e.g. miR-127) [30, 38, 39] given previous results were mainly obtained using mouse or rat models and, in the case of miR-127, showed conflicting features [40, 41].

As a marker of local tissue apoptosis, we found increased levels of cleaved caspase-3 subunits in both the moderate and severe trauma groups at the ipsilateral control but not at the contusion site or the contralateral control. Following multiple-trauma, caspase-3 levels in lung tissue samples have previously been reported to increase within few hours [42]. In the present study, lung tissue was sampled not before 48 hours, which may have prevented detection of early caspase-3 dynamics and account for differences between right and left lung. Previous studies addressed the role of lung apoptosis in the development of posttraumatic ARDS by either delaying neutrophil apoptosis and/or by enhancement of epithelial cell apoptosis [43, 44]. Matute-Bello et al. reported increased concentrations of soluble Fas ligand in BAL samples from patients with ARDS, which in turn induced apoptosis in healthy lung epithelial cells in vitro [45]. The effects of hypothermia on the posttraumatic apoptosis include the inhibition of the initiation of the apoptotic cascades (e.g. via reduced cJun N-terminal kinase activation) and the preservation of mitochondrial function [46, 47].

Therapeutic intervention with controlled, mild hypothermia has long been controversial as a treatment option for trauma patients and is currently not recommended by international guidelines. While numerous experimental studies in animal models reported promising results (e.g., a decrease in TNF-α release and a delay in IL-1 and IL-6 release, increased concentrations of interleukin-10, and increased survival after hemorrhagic shock were shown after intervention with hypothermia) [46, 48, 49], the mechanisms by which hypothermia influences the posttraumatic immune response after multiple trauma proofed difficult to unravel. Additionally, hypothermia can affect trauma-induced coagulopathy by impaired clotting factor activity and platelet function [50–52]. However, data from a study of 310 multiple-trauma patients found that accidental hypothermia was not an independent risk factor for post-traumatic complications (e.g. sepsis, MODS) [53].

By contrast, therapeutic hypothermia is an established treatment option in the care of survivors of out-of-hospital cardiac arrest [54]. While there is evidence that hypothermia improves neurological outcome [54] and survival [55], early-onset pneumonia has been identified as a common complication of cooling after successful cardio-pulmonary resuscitation [56, 57]. In a recent meta-analysis including 23 studies, Geurts et al. found therapeutic hypothermia to increase the risk of pneumonia and sepsis [58]. In contrast, recently reported results from the randomized controlled TTM2 trial reported consistent frequencies of adverse effects (e.g. pneumonia or sepsis) [59]. Mechanistically, the immune modulation by therapeutic hypothermia suppresses the release of pro-inflammatory cytokines which mediate the activation of the innate and adaptive immune response [60]. Hence, patients who have been exposed to severe stress (e.g. multiple trauma or cardiac arrest with cardiopulmonary resuscitation) are susceptible to pulmonary infection, to which treatment with mild hypothermia may further contribute [61].

In our study, following the intervention with mild hypothermia, we observed decreasing levels of pro-inflammatory IL-6 while concentrations of anti-inflammatory IL-10 were increasing. While these trends were only observed in the severe trauma group and did not reach statistical significance, they are in agreement with previously reported findings [26] and experimental studies [48, 62]. The anti-inflammatory effects of induced hypothermia are known to occur during the late posttraumatic phase and support the increased risk for infectious complications (e.g. pneumonia or sepsis).

Taken together, our findings show no substantial benefit of the intervention with mild hypothermia over normothermic control groups. While the risk for adverse effects such as a prolonged immunologic imbalance might result in posttraumatic complications (e.g. infection, sepsis, MODS), we found no beneficial effect of mild hypothermia treatment in our investigation.

The purpose of our study was to investigate the pulmonary inflammatory response to multiple trauma, including hemorrhagic shock, in a clinically relevant porcine model over 48 hours and to gain insight into whether an intervention with mild therapeutic hypothermia would attenuate the respiratory inflammatory response. Due to the very specific injury pattern and severity of our model, our findings only apply to a specific group of patients. Thus, conclusions might not apply to other patient populations (e.g. post-surgery, cardiac arrest). Given that most biomarkers were sampled after 48 hours, we may have missed an initial early change followed by return to homeostasis. As with all animal models, our data is subject to certain restrictions: Although porcine models have great benefits for immunologic research, the inflammatory response may not be identical and inter-species differences have to be taken into account [63]. Furthermore, the coagulation system in pigs is different compared to humans [64], and hypothermia (accidental or therapeutic) may affect the physiologic cascade [65]. We did not include survival as an outcome parameter. Due to i) the small sample size granted by authorities, ii) (partly) small effect sizes, iii) (partly) large variability, and iv) the need to use non-parametric testing, there is a possibility of false negative statistical results.

In conclusion, we observed signs of a time-dependent activation of the local and trauma-distant pulmonary inflammatory response in a porcine model of combined trauma (blunt chest trauma, liver laceration, and tibia fracture) and severe hemorrhagic shock. Intervention with mild, therapeutic hypothermia had no considerable effect during the first 48 hours after trauma.

## Supporting information

S1 Fig Caspase-3 western blot. Western blot of pro- and cleaved caspase-3 [ratio to ß-actin]. Shown are representative blots separated by location of lung tissue sampling (contusion site, ipsilateral control, and contralateral control). Lung tissue samples were homogenized, separated, blotted and membranes were cut horizontally along 38 kDa to enable simultaneous analysis of ß-actin (40 kDa), pro-caspase-3 (32 kDa) and cleaved caspase-3 subunits (11, 17, 20 kDa). Untreated and cytochrome c-treated Jurkat control cell extracts served as controls. Statistical analysis based on a quantification of pro- and cleaved caspase-3 protein levels (ratio to ß-actin) applying a Wilcoxon matched-pairs signed rank test is shown in S9 Table in S1 File and S2 Fig.
(TIF)

S2 Fig Apoptosis in lung tissue. Western blot quantification of pro- and cleaved caspase-3 protein levels [ratio to ß-actin]. Lung tissue samples from the contusion site (right lower lobe), the ipsilateral control (right upper lobe) and the contralateral control (left upper lobe) were homogenized, separated, blotted, incubated and quantified by densitometry. Mean values+SD on linear scale are shown. Statistical analysis: Wilcoxon matched-pairs signed rank test: *p<0.05, **p<0.01.
(TIF)

S1 File (DOCX)
