# Supplementary figures and images for: Pulmonary inflammatory response and immunomodulation to multiple trauma and hemorrhagic shock in pigs
Source: PLoS One. 2022 Dec 7;17(12):e0278766. doi: 10.1371/journal.pone.0278766 (PMC9728855; doi:10.1371/journal.pone.0278766)

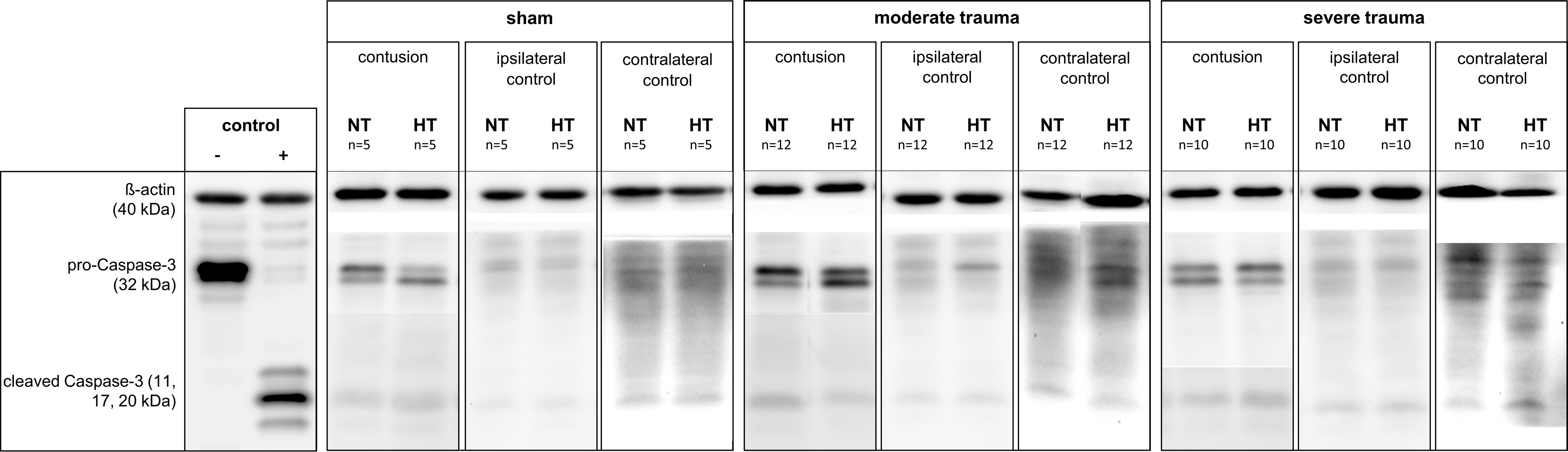

Supplement: S1 Fig — Western blot of pro- and cleaved caspase-3 [ratio to ß-actin]. Shown are representative blots separated by location of lung tissue sampling (contusion site, ipsilateral control, and contralateral control). Lung tissue samples were homogenized, separated, blotted and membranes were cut horizontally along 38 kDa to enable simultaneous analysis of ß-actin (40 kDa), pro-caspase-3 (32 kDa) and cleaved caspase-3 subunits (11, 17, 20 kDa). Untreated and cytochrome c-treated Jurkat control cell extracts served as controls. Statistical analysis based on a quantification of pro- and cleaved caspase-3 protein levels (ratio to ß-actin) applying a Wilcoxon matched-pairs signed rank test is shown in S9 Table in S1 File and S2 Fig. (TIF) [file pone.0278766.s001.tif]

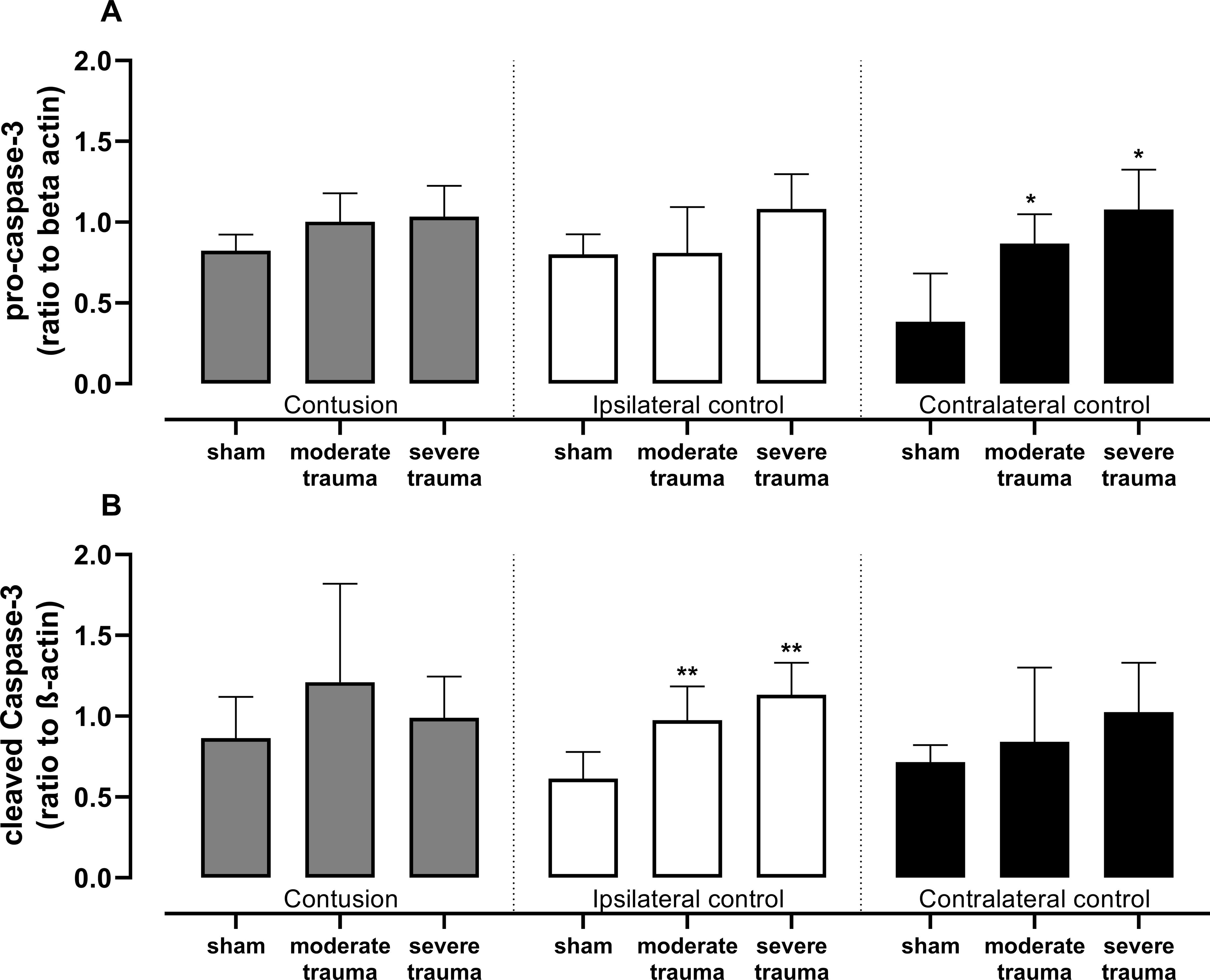

Supplement: S2 Fig — Western blot quantification of pro- and cleaved caspase-3 protein levels [ratio to ß-actin]. Lung tissue samples from the contusion site (right lower lobe), the ipsilateral control (right upper lobe) and the contralateral control (left upper lobe) were homogenized, separated, blotted, incubated and quantified by densitometry. Mean values+SD on linear scale are shown. Statistical analysis: Wilcoxon matched-pairs signed rank test: *p<0.05, **p<0.01. (TIF) [file pone.0278766.s002.tif]
