# Supplementary material for: Pulmonary inflammatory response and immunomodulation to multiple trauma and hemorrhagic shock in pigs
Source: PLoS One. 2022 Dec 7;17(12):e0278766. doi: 10.1371/journal.pone.0278766 (PMC9728855; doi:10.1371/journal.pone.0278766)
Supplement: S1 File — (DOCX) [file pone.0278766.s003.docx]

**Pulmonary inflammatory response and immunomodulation to multiple trauma and hemorrhagic shock in pigs**

**- Online supplement -**

Marc-Alexander Oestreich, Kerstin Seidel, Wilhelm Bertrams, Hans-Helge Müller, Martin Sassen, Thorsten Steinfeldt, Hinnerk Wulf, Bernd Schmeck

**Statistical analysis**

The primary outcome of our study was the characterization of the pulmonary inflammatory response (quantified by BAL fluid levels of cytokines IL-6 and IL-10, changes in gene-expression of IL-1, IL-6, IL-8, SP-C, IKBA, TXN, and COX-2 and miRNA-146 and -155, as well as tissue caspase-3 levels) to multiple trauma and hemorrhagic shock under normothermic conditions. We hypothesized that the induction of combined trauma and hemorrhagic shock would induce a pronounced inflammatory response that would allow the characterization of the underlying pathophysiological mechanisms.

Secondary outcomes were i) the characterization of immunomodulatory effects of mild hypothermia on cytokine-levels, gene and microRNA expression, and lung tissue apoptosis compared to normothermia (we hypothesized that there would be differences in cytokine levels (BAL fluid) and gene expression (lung tissue samples) between the study groups.), and ii) the characterization of a systemic activation of inflammatory response regulators (microRNA expression) and markers of lung tissue apoptosis (caspase-3) in the non-injured left lung and the contused right lung (we hypothesized that there would be remote organ inflammation and ongoing apoptosis in the contralateral, non-injured lung).

The minimal dataset can be accessed here: <https://doi.org/10.6084/m9.figshare.21324561.v1>. Original western blot images are available: <https://doi.org/10.6084/m9.figshare.21324606.v4>.

BAL fluid

Adjusted BAL4 values ($\frac{logBAL4}{logBAL1}$) of the trauma groups (sham, moderate trauma, and severe trauma) were compared by a closed-testing principle to reduce the number of tests required[^1^](#_ENREF_1). First, we performed a Kruskal-Wallis test (sham, moderate trauma, severe trauma; p≤0.05) followed by a Wilcoxon-Mann-Whitney test (sham vs. moderate trauma, sham vs. severe trauma, and moderate vs. severe trauma; p≤0.05), if the global Kruskal-Wallis test was significant.

Adjusted BAL4 values ($\frac{logBAL4}{logBAL1}$) of the treatment groups (normothermia (NT) and hypothermia (HT)) were compared by a Wilcoxon-Mann-Whitney test (HT_moderate trauma_ vs. NT_moderate trauma_ and HT_severe trauma_ vs. NT_severe trauma_; p≤0.05). Additionally, we investigated the interaction terms for the interaction of trauma (moderate and severe trauma groups combined) and sham based on the variance ($s^{2}=\frac{\sum{(x-\bar{x})}^{2}}{n-1}$) of log-transformed adjusted BAL4 values ($\frac{logBAL4}{logBAL1}$). Due to the very low number of observations, we report the interaction terms without further statistical analysis.

Lung tissue

Lung tissue homogenates from the right upper lobe (ipsilateral control), right lower lobe (contusion site), and left upper lobe (contralateral site) were analyzed by RT-qPCR and logarithmic data were normalized (to β-actin for gene expression, to microRNA-17 for microRNA expression). Gene and microRNA expression between the trauma groups (sham, moderate trauma, and severe trauma) were compared by a closed-testing principle (see above). Gene and microRNA expression between the treatment groups (NT and HT) were compared by a Wilcoxon-Mann-Whitney test (HT_moderate trauma_ vs. NT_moderate trauma_ and HT_severe trauma_ vs. NT_severe trauma_; p≤0.05) with interaction terms for the interaction of trauma (moderate and severe trauma groups combined) and sham. Additionally, the microRNA expression and lung tissue apoptosis (caspase-3 western blot) were compared between the non-injured left lung (contralateral control) and the contused right lung (ipsilateral control) by a Wilcoxon matched-pairs signed rank test.

**S1 Table: Primer sequences**

| β-actin | fwd. | 5’-AAGGACCTCTACGCCAACAC-3’ |
| --- | --- | --- |
| [*actb*] | rev. | 5’-ACACGGAGTACTTGCGCTCT-3’ |
| Interleukin-1 beta | fwd. | 5’-GCACAAAGGCCATTCAGGGA-3’ |
| [*il1b1*] | rev. | 5’-GGCGGGTTCAGGTACTATGG-3’ |
| Interleukin-6 | fwd. | 5’-CCACCAGGAACGAAAGAGAG-3’ |
| [*il6*] | rev. | 5’-GTTTTGTCCGGAGAGGTGAA-3’ |
| Interleukin-8 | fwd. | 5’-CCAAACTGGCTGTTGCCTTC-3’ |
| [*cxcl8*] | rev. | 5’-TGCACTGGCATCGAAGTTCT-3’ |
| Surfactant protein C | fwd. | 5’-CTTGTCGTCGTGGTGATTGT-3’ |
| [*sftpc*] | rev. | 5’-AGGAGCCGCTGGTAGTCATA-3’ |
| IKBA | fwd. | 5’-TGTCTTTGGGTGCTGATGTC-3’ |
| [*nfkbia*] | rev. | 5’-ACTCGGATACAGCAGCAGCT-3’ |
| Thioredoxin | fwd. | 5’-GGTGTGGGCCTTGCAAAATG-3’ |
| [*txn*] | rev. | 5’-GGAAGGTTGGCATGCATTTGA-3’ |
| Cyclooxygenase 2 | fwd. | 5’-AGATCAGAAGCGAGGACCAG-3’ |
| [*cox2*] | rev. | 5’-GTGTGAGGCGGGTAGATCAT-3’ |
| miroRNA-17 | fwd. | 5’-CAAAGTGCTTACAGTGCAG-3’ |
| [*ssc-miR-17-5p*] | rev. | 5’-GGTCCAGTTTTTTTTTTTTTTTCTAC-3’ |
| miroRNA-146a | fwd. | 5’-CAGTGAGAACTGAATTCCATG-3’ |
| [*ssc-miR-146a-5p*] | rev. | 5’-GGTCCAGTTTTTTTTTTTTTTTAACC-3’ |
| miroRNA-155 | fwd. | 5’-CGCAGTTAATGCTAATTGTG-3’ |
| [*ssc-miR-155*] | rev. | 5’-CAGTTTTTTTTTTTTTTTCCCCTATC-3’ |

Primer sequences for RT-qPCR analysis. fwd: forward; rev: reverse.

**S2 Table: Time dependent activation of the pulmonary inflammatory response under normothermic conditions in BAL fluid**

|  |  | **BAL 1** | | | **BAL 2** | | | **BAL 3** | | | **BAL 4** | | | **adjusted BAL 4** | | |
| --- | --- | --- | --- | --- | --- | --- | --- | --- | --- | --- | --- | --- | --- | --- | --- | --- |
| **Cytokine** | **Trauma** | **n** | **mean** | **SD** | **n** | **mean** | **SD** | **n** | **mean** | **SD** | **n** | **mean** | **SD** | **p value*** | **p value^†^** | **p value^‡^** |
| IL-6 | sham | 4 | 0.10 | 0.02 | 4 | 0.23 | 0.16 | 3 | 0.17 | 0.10 | 5 | 0.32 | 0.32 | **0.037** | - |  |
|  | moderate | 8 | 0.17 | 0.12 | 11 | 0.11 | 0.10 | 11 | 0.28 | 0.19 | 11 | 0.29 | 0.23 | - | 0.743 | **0.016** |
|  | severe | 9 | 0.24 | 0.25 | 9 | 0.37 | 0.23 | 8 | 0.44 | 0.36 | 9 | 0.90 | 0.51 | - | 0.083 |  |
| IL-10 | sham | 0 | 0.00 | - | 2 | 0.02 | 0.00 | 2 | 0.02 | 0.00 | 1 | 0.02 | - | 0.194 | - | - |
|  | moderate | 1 | 0.03 | - | 1 | 0.00 | - | 4 | 0.01 | 0.00 | 5 | 0.03 | 0.03 | - | - | - |
|  | severe | 4 | 0.04 | 0.02 | 5 | 0.03 | 0.02 | 4 | 0.01 | 0.01 | 4 | 0.05 | 0.02 | - | - | - |

Bronchoalveolar lavage fluid was sampled at four time points (before and after trauma, after 24 and 48 hours), concentrated, and analyzed by ELISA [pg cytokine/µg total protein]. The number of observations (n), mean, and standard deviation (SD) for each timepoint are shown. Statistical analysis: Closed-testing principle of adjusted BAL4 values ($\frac{logBAL4}{logBAL1}$) among the trauma groups: *Kruskal-Wallis test (*p≤0.05), ^†^Wilcoxon-Mann-Whitney (sham vs. moderate trauma, sham vs. severe trauma; ^†^p≤0.05), ^‡^Wilcoxon-Mann-Whitney (moderate trauma vs. severe trauma; ^‡^p≤0.05). Significant p-values (p≤0.05) in bold print.

**S3 Table: Effects of mild hypothermia on cytokine levels in BAL fluid**

| **A)** |  |  | | |  | | |  | | |
| --- | --- | --- | --- | --- | --- | --- | --- | --- | --- | --- |
|  |  | **Normothermia** | | | **Hypothermia** | | | **Difference** | | |
| **Cytokine** | **Trauma** | **n** | **mean** | **SD** | **n** | **mean** | **SD** | **mean** | **95% CI** | **p value** |
| IL-6 | moderate | 11 | 0.13 | 0.28 | 15 | 0.44 | 0.82 | 0.31 | -0.13 to 0.67 | 0.217 |
|  | severe | 9 | 0.51 | 0.54 | 8 | 0.53 | 0.49 | 0.02 | -0.70 to 0.45 | 0.370 |
| IL-10 | moderate | 5 | 0.01 | 0.04 | 5 | 0.04 | 0.02 | 0.03 | -0.05 to 0.05 | 0.222 |
|  | severe | 4 | 0.01 | 0.03 | 5 | 0.07 | 0.06 | 0.06 | -0.03 to 0.16 | 0.886 |

| **B)** | |  | |  | |  |  | |  | |  | |
| --- | --- | --- | --- | --- | --- | --- | --- | --- | --- | --- | --- | --- |
| **Cytokine** | **Trauma** | | **Hypothermia** | | **Normothermia** | | | **Difference*** | | **Difference^†^** | | **Interaction term** |
| IL-6 | trauma | | 0.99 | | 0.56 | | | 0.43 | | 1.54 | | 1.54 - 0.76 |
|  | sham | | 0.27 | | 0.53 | | | -0.26 | | 0.77 | |  |
| IL-10 | trauma | | 1.78 | | 3.15 | | | -1.37 | | 0.25 | | 0.25 - 1.95 |
|  | sham | | 0.67 | | - | | | 0.67 | | 1.95 | |  |

Bronchoalveolar lavage fluid was sampled at four time points (before and after trauma, after 24 and 48 hours), concentrated, and analyzed by ELISA [pg cytokine/µg total protein]. Table A) shows the number of observations (n), mean, and standard deviation (SD) for both trauma groups under normothermic and hypothermic conditions. Statistical analysis: Wilcoxon-Mann-Whitney test (p≤0.05). Table B) shows the interaction terms for the interaction of the combined trauma groups (without further statistical analysis). Logarithmic (*) and exponentiated (^†^) differences in variance are shown.

**S4 Table: Multiple trauma and hemorrhagic shock induce a transcriptional immune response**

|  |  | **Ipsilateral control (right upper lobe)** | | | | | | **Contusion (right lower lobe)** | | | | | | **Contralateral control (left upper lobe)** | | | | | |
| --- | --- | --- | --- | --- | --- | --- | --- | --- | --- | --- | --- | --- | --- | --- | --- | --- | --- | --- | --- |
| **Gene** | **Trauma** | **n** | **mean** | **SD** | **p value*** | **p value^†^** | **p value^‡^** | **n** | **mean** | **SD** | **p value*** | **p value^†^** | **p value^‡^** | **n** | **mean** | **SD** | **p value*** | **p value^†^** | **p value^‡^** |
| IL-1β | sham | 4 | 0.96 | 0.20 | **0.001** | - | - | 5 | 2.01 | 1.77 | 0.605 | - | - | 5 | 1.21 | 0.92 | 0.604 | - | - |
| [*il1b1*] | moderate | 7 | 3.65 | 2.91 | - | **0.012** | **0.014** | 7 | 2.99 | 2.52 | - | - | - | 8 | 2.45 | 1.88 | - | - | - |
|  | severe | 6 | 45.91 | 42.72 | - | **0.010** |  | 6 | 2.89 | 2.70 | - | - | - | 6 | 2.71 | 2.42 | - | - | - |
| IL-6 | sham | 4 | 0.97 | 0.26 | **0.006** | - | - | 4 | 0.80 | 0.20 | **0.012** | - | - | 4 | 0.74 | 0.34 | 0.136 | - | - |
| [*il6*] | moderate | 8 | 7.64 | 7.29 | - | **0.028** | 0.127 | 7 | 9.16 | 8.67 | - | **0.012** | 0.181 | 6 | 2.27 | 2.39 | - | - | - |
|  | severe | 5 | 31.73 | 34.04 | - | **0.016** |  | 6 | 3.68 | 2.45 | - | 0.067 |  | 6 | 1.58 | 1.16 | - | - | - |
| IL-8 | sham | 4 | 2.72 | 1.10 | **0.003** | - | - | 4 | 1.88 | 1.22 | **0.012** | - | - | 5 | 1.34 | 0.65 | 0.523 | - | - |
| [*cxcl8*] | moderate | 8 | 12.14 | 14.27 | - | 0.999 | **0.005** | 7 | 12.46 | 11.57 | - | **0.006** |  | 7 | 2.67 | 2.36 | - | - | - |
|  | severe | 6 | 136.70 | 84.31 | - | **0.010** |  | 6 | 8.60 | 8.09 | - | **0.038** |  | 6 | 6.06 | 5.61 | - | - | - |
| COX-2 | sham | 4 | 1.74 | 1.14 | **0.008** | - | - | 5 | 0.94 | 0.32 | 0.814 | - | - | 5 | 1.85 | 0.63 | 0.452 | - | - |
| [*cox2*] | moderate | 9 | 1.92 | 0.84 | - | 0.825 | **0.008** | 11 | 0.90 | 0.25 | - | - | - | 11 | 2.25 | 0.77 | - | - | - |
|  | severe | 8 | 3.64 | 1.44 | - | **0.028** |  | 8 | 0.99 | 0.32 | - | - | - | 8 | 2.42 | 0.67 | - | - | - |
| IκBα | sham | 5 | 1.28 | 0.56 | 0.106 | - | - | 5 | 1.30 | 0.26 | 0.557 | - | - | 5 | 0.92 | 0.28 | 0.325 | - | - |
| [*nfkbia*] | moderate | 8 | 1.12 | 0.58 | - | - | - | 8 | 1.86 | 1.16 | - | - | - | 8 | 1.18 | 0.51 | - | - | - |
|  | severe | 6 | 3.18 | 1.87 | - | - | - | 7 | 1.65 | 0.73 | - | - | - | 6 | 1.34 | 0.50 | - | - | - |
| TXN | sham | 5 | 1.74 | 0.94 | 0.361 | - | - | 5 | 1.21 | 0.29 | 0.064 | - | - | 4 | 1.00 | 0.02 | 0.156 | - | - |
| [*txn*] | moderate | 8 | 1.45 | 1.02 | - | - | - | 8 | 1.67 | 0.89 | - | - | - | 8 | 1.05 | 0.21 | - | - | - |
|  | severe | 5 | 1.98 | 0.48 | - | - | - | 7 | 1.95 | 0.45 | - | - | - | 6 | 1.71 | 1.24 | - | - | - |
| SP-C | sham | 5 | 1.00 | 0.39 | 0.842 | - | - | 5 | 0.61 | 0.35 | 0.312 | - | - | 5 | 0.77 | 0.20 | 0.131 | - | - |
| [*sftpc*] | moderate | 12 | 1.07 | 0.78 | - | - | - | 12 | 0.61 | 0.37 | - | - | - | 12 | 1.12 | 0.47 | - | - | - |
|  | severe | 8 | 0.86 | 0.61 | - | - | - | 8 | 0.86 | 0.39 | - | - | - | 8 | 1.34 | 0.62 | - | - | - |

Gene expression [x-fold] of pro-inflammatory biomarkers Interleukin-1β (*il1b1*), Interleukin-6 (*il6*), Interleukin-8 (*cxcl8*), and Cyclooxygenase-2 (*cox2*) and anti-inflammatory IκBα (*nfkbia*), Thioredoxin (*txn*), and Surfactant-associated protein C (*sftpc*) measured in lung tissue samples from the right upper lobe (ipsilateral control), the right lower lobe (contusion site) and the left upper lobe (contralateral control) were analyzed by RT-qPCR and normalized to beta-actin. The number of observations (n), mean, standard deviation (SD) and corresponding p-values are shown. Statistical analysis: Closed-testing principle among the trauma groups. *Kruskal-Wallis test (*p≤0.05), ^†^Wilcoxon-Mann-Whitney test (sham vs. moderate trauma, sham vs. severe trauma; ^†^p≤0.05), ^‡^Wilcoxon-Mann-Whitney test (moderate trauma vs. severe trauma; ^‡^p≤0.05). Significant p-values (p≤0.05) in bold print.

**S5 Table: Effects of mild hypothermia on the transcriptional immune response**

| **A)** |  |  | | |  | | |  | | |
| --- | --- | --- | --- | --- | --- | --- | --- | --- | --- | --- |
|  |  | **Normothermia** | | | **Hypothermia** | | | **Difference** | | |
| **Gene** | **Trauma** | **n** | **median** | **SD** | **n** | **median** | **SD** | **mean** | **95% CI** | **p value** |
| IL-1β | moderate | 7 | 2.42 | 2.91 | 11 | 4.31 | 8.25 | 1.89 | -1.7 to 12.0 | 0.328 |
| [*il1b1*] | severe | 6 | 35.97 | 42.72 | 6 | 23.41 | 41.14 | -12.56 | -79.3 to 64.1 | 0.485 |
| IL-6 | moderate | 8 | 5.96 | 7.29 | 11 | 8.41 | 8.10 | 2.46 | -2.9 to 10.7 | 0.351 |
| [*il6*] | severe | 5 | 18.14 | 34.04 | 6 | 21.04 | 34.96 | 2.90 | -50.8 to 60.1 | 0.931 |
| IL-8 | moderate | 8 | 5.11 | 14.27 | 10 | 25.29 | 47.19 | 20.18 | -2.1 to 57.2 | 0.122 |
| [*cxcl8*] | severe | 6 | 133.70 | 84.31 | 6 | 72.51 | 148.60 | -61.21 | -223.5 to 147.4 | 0.589 |
| COX-2 | moderate | 9 | 1.77 | 0.84 | 11 | 2.02 | 0.62 | 0.25 | -0.7 to 0.9 | 0.412 |
| [*cox2*] | severe | 8 | 3.62 | 1.44 | 8 | 4.11 | 3.64 | 0.49 | -1.8 to 4.5 | 0.574 |
| IκBα | moderate | 8 | 1.27 | 0.58 | 11 | 1.96 | 1.08 | 0.69 | -0.2 to 1.7 | 0.109 |
| [*nfkbia*] | severe | 6 | 3.21 | 1.87 | 7 | 1.95 | 1.79 | -1.27 | -3.1 to 2.0 | 0.731 |
| TXN | moderate | 8 | 1.21 | 1.02 | 10 | 1.11 | 0.22 | -0.09 | -1.1 to 0.4 | 0.633 |
| [*txn*] | severe | 5 | 2.03 | 0.48 | 6 | 1.82 | 1.69 | -0.21 | -0.8 to 3.6 | 0.429 |
| SP-C | moderate | 12 | 1.07 | 0.78 | 12 | 0.82 | 0.34 | -0.25 | -0.6 to 0.3 | 0.755 |
| [*sftpc*] | severe | 8 | 0.73 | 0.61 | 8 | 0.60 | 0.77 | -0.13 | -0.6 to 0.8 | 0.959 |

| **B)** |  |  |  |  |  |  |  |  |  |  |  |  |
| --- | --- | --- | --- | --- | --- | --- | --- | --- | --- | --- | --- | --- |
| **Gene** |  | **Trauma** |  | **Hypothermia** |  | **Normothermia** |  | **Difference*** |  | **Difference^†^** |  | **Interaction term** |
| IL-1β |  | trauma |  | 1.70 |  | 0.61 |  | 1.09 |  | 2.97 |  | 2.97 - 1.51 |
| [*il1b1*] |  | sham |  | 0.45 |  | 0.04 |  | 0.41 |  | 1.51 |  |  |
| IL-6 |  | trauma |  | 2.04 |  | 1.21 |  | 0.83 |  | 2.29 |  | 2.29 - 1.39 |
| [*il6*] |  | sham |  | 0.43 |  | 0.10 |  | 0.33 |  | 1.39 |  |  |
| IL-8 |  | trauma |  | 3.02 |  | 1.61 |  | 1.41 |  | 4.10 |  | 4.10 - 0.92 |
| [*cxcl8*] |  | sham |  | 0.25 |  | 0.34 |  | -0.09 |  | 0.92 |  |  |
| COX-2 |  | trauma |  | 0.43 |  | 0.36 |  | 0.07 |  | 1.08 |  | 1.08 - 1.05 |
| [*cox2*] |  | sham |  | 0.30 |  | 0.25 |  | 0.05 |  | 1.05 |  |  |
| IκBα |  | trauma |  | 0.24 |  | 0.60 |  | -0.36 |  | 0.70 |  | 0.70 - 0.93 |
| [*nfkbia*] |  | sham |  | 0.05 |  | 0.12 |  | -0.07 |  | 0.93 |  |  |
| TXN |  | trauma |  | 0.13 |  | 0.25 |  | -0.13 |  | 0.88 |  | 0.88 - 1.42 |
| [*txn*] |  | sham |  | 0.41 |  | 0.06 |  | 0.35 |  | 1.42 |  |  |
| SP-C |  | trauma |  | 0.47 |  | 0.51 |  | -0.05 |  | 0.96 |  | 0.96 - 1.06 |
| [*sftpc*] |  | sham |  | 0.31 |  | 0.25 |  | 0.06 |  | 1.06 |  |  |

Lung tissue homogenates from the right upper lobe (ipsilateral control) were analyzed by RT-qPCR, logarithmic data were normalized to β-actin. Table A) shows the number of observations (n), mean, and standard deviation (SD) for both trauma groups under normothermic and hypothermic conditions. Statistical analysis: Wilcoxon-Mann-Whitney test (p≤0.05). Table B) shows the interaction terms for the interaction of the combined trauma groups (without further statistical analysis). Logarithmic (*) and exponentiated (^†^) differences in variance are shown

**S6 Table: Trauma-distant activation of inflammatory response regulators in** **the non-injured lung**

| **A)** |  |  |  |  |  |  |  |  |  |  |  |  |  |  |  |  |  |  |  |  |  |
| --- | --- | --- | --- | --- | --- | --- | --- | --- | --- | --- | --- | --- | --- | --- | --- | --- | --- | --- | --- | --- | --- |
| **miRNA** | **Trauma** | **Ipsilateral control (**right upper lobe**)** | | | | | |  | **Contusion (**right lower lobe**)** | | | | | |  | **Contralateral control (**left upper lobe**)** | | | | | |
|  |  | **n** | **mean** | **SD** | **p-value*** | **p value^†^** | **p value^‡^** |  | **n** | **mean** | **SD** | **p-value*** | **p value^†^** | **p value^‡^** |  | **n** | **mean** | **SD** | **p-value*** | **p value^†^** | **p value^‡^** |
| 146a | sham | 5 | 1.08 | 0.26 | 0.065 | - | - |  | 5 | 1.18 | 0.56 | 0.510 | - | - |  | 5 | 1.45 | 0.63 | 0.228 | - | - |
|  | moderate | 10 | 0.81 | 0.35 | **-** | **-** | **-** |  | 10 | 1.01 | 0.30 | **-** | **-** | **-** |  | 11 | 1.48 | 0.63 | **-** | **-** | **-** |
|  | severe | 8 | 1.11 | 0.26 | **-** | **-** | **-** |  | 8 | 1.30 | 0.49 | **-** | **-** | **-** |  | 8 | 1.92 | 1.02 | **-** | **-** | **-** |
| 155 | sham | 5 | 1.04 | 0.10 | 0.582 | - | - |  | 5 | 1.09 | 0.25 | 0.723 | - | - |  | 3 | 1.06 | 0.07 | 0.155 | - | - |
|  | moderate | 11 | 0.93 | 0.27 | **-** | **-** | **-** |  | 11 | 1.06 | 0.23 | **-** | **-** | **-** |  | 9 | 1.06 | 0.28 | **-** | **-** | **-** |
|  | severe | 8 | 0.97 | 0.18 | **-** | **-** | **-** |  | 8 | 1.22 | 0.39 | **-** | **-** | **-** |  | 7 | 1.26 | 0.21 | **-** | **-** | **-** |

| **B)** |  |  |  |  |  |  |  |  |  |  |  |  |  |  |
| --- | --- | --- | --- | --- | --- | --- | --- | --- | --- | --- | --- | --- | --- | --- |
|  |  |  |  | **Ipsilateral control** | | |  | **Contralateral control** | | |  | **Difference** | | |
| **miRNA** |  | **Trauma** |  | **n** | **mean** | **SD** |  | **n** | **mean** | **SD** |  | **mean** | **95% CI** | **p value** |
| 146a |  | sham |  | 5 | 1.08 | 0.26 |  | 5 | 1.45 | 0.63 |  | 0.37 | -0.2 to 1.0 | 0.250 |
|  |  | moderate |  | 10 | 0.81 | 0.35 |  | 11 | 1.48 | 0.63 |  | 0.63 | 0.4 to 0.9 | **0.002** |
|  |  | severe |  | 8 | 1.11 | 0.26 |  | 8 | 1.92 | 1.02 |  | 0.82 | 0.1 to 1.5 | **0.008** |
| 155 |  | sham |  | 5 | 1.04 | 0.10 |  | 3 | 1.06 | 0.07 |  | -0.04 | -0.2 to 0.1 | 0.500 |
|  |  | moderate |  | 11 | 0.93 | 0.27 |  | 9 | 1.06 | 0.28 |  | 0.19 | 0.1 to 0.3 | **0.027** |
|  |  | severe |  | 8 | 0.97 | 0.18 |  | 7 | 1.26 | 0.21 |  | 0.33 | 0.1 to 0.5 | **0.016** |

Expression of microRNA-146a and microRNA-155 [x-fold]. Lung tissue homogenates from the right lower lobe (contusion), the right upper lobe (ipsilateral control) and the non-injured left upper lobe (contralateral control) were analyzed by RT-qPCR and logarithmic data were normalized to microRNA-17. Table A) shows the number of observations (n), mean, standard deviation (SD) and corresponding p-values. Statistical analysis: Closed-testing principle among the trauma groups. *Kruskal-Wallis test (*p≤0.05), ^†^Wilcoxon-Mann-Whitney test (sham vs. moderate trauma, sham vs. severe trauma; ^†^p≤0.05), ^‡^Wilcoxon-Mann-Whitney test (moderate trauma vs. severe trauma; ^‡^p≤0.05). Significant p-values (p≤0.05) in bold print. Table B) shows the number of observations (n), mean, standard deviation (SD) and corresponding p-values between the injured right lung and the uninjured (sham) left lung. Statistical analysis: Wilcoxon matched-pairs signed rank test (p≤0.05). Significant p-values (p≤0.05) in bold print.

**S7 Table: Effects of mild hypothermia on inflammatory response regulators**

| **A)** |  |  |  |  |  |  |  |  |  |  |  |  |  |
| --- | --- | --- | --- | --- | --- | --- | --- | --- | --- | --- | --- | --- | --- |
|  |  |  | **Normothermia** | | |  | **Hypothermia** | | |  | **Difference** | | |
| **miRNA** | **Trauma** |  | **n** | **median** | **SD** |  | **n** | **median** | **SD** |  | **mean** | **95% CI** | **p value** |
| 146a | moderate |  | 10 | 0.68 | 0.35 |  | 9 | 0.79 | 0.24 |  | 0.10 | -0.3 to 0.3 | 0.842 |
|  | severe |  | 8 | 1.11 | 0.26 |  | 8 | 1.18 | 0.48 |  | 0.08 | -0.2 to 0.5 | 0.505 |
| 155 | moderate |  | 11 | 0.85 | 0.27 |  | 12 | 0.93 | 0.30 |  | 0.08 | -0.2 to 0.3 | 0.786 |
|  | severe |  | 8 | 0.97 | 0.18 |  | 7 | 0.94 | 0.20 |  | -0.03 | -0.4 to 0.1 | 0.463 |

| **B)** |  |  |  |  |  |  |  |  |  |  |
| --- | --- | --- | --- | --- | --- | --- | --- | --- | --- | --- |
| **miRNA** | **Trauma** |  | **Hypothermia** |  | **Normothermia** |  | **Difference*** | **Difference^†^** |  | **Interaction term** |
| 146a | trauma |  | 0.15 |  | 0.19 |  | -0.04 | 0.96 |  | 0.96 - 0.95 |
|  | sham |  | 0.09 |  | 0.14 |  | -0.05 | 0.95 |  |  |
| 155 | trauma |  | 0.09 |  | 0.07 |  | 0.02 | 1.02 |  | 1.02 - 1.08 |
|  | sham |  | 0.10 |  | 0.02 |  | 0.08 | 1.08 |  |  |

Lung tissue homogenates from the right upper lobe (ipsilateral control) were analyzed by RT-qPCR, normalized to microRNA-17, and log-transformed. Lung tissue homogenates from the right upper lobe (ipsilateral control) were analyzed by RT-qPCR and logaritihmic data were normalized to β-actin. Table A) shows the number of observations (n), mean, and standard deviation (SD) for both trauma groups under normothermic and hypothermic conditions. Statistical analysis: Wilcoxon-Mann-Whitney test (p≤0.05). Table B) shows the interaction terms for the interaction of the combined trauma groups (without further statistical analysis). Logarithmic (*) and exponentiated (^†^) differences in variance are shown.

S8 Table: Apoptosis in lung tissue

| **Protein** | **Trauma** | **Ipsilateral control** (right upper lobe) | | | | | | **Contusion** (right lower lobe) | | | | | | **Contralateral control** (left upper lobe) | | | | | |
| --- | --- | --- | --- | --- | --- | --- | --- | --- | --- | --- | --- | --- | --- | --- | --- | --- | --- | --- | --- |
|  |  | **n** | **mean** | **SD** | **p-value*** | **p value^†^** | **p value^‡^** | **n** | **mean** | **SD** | **p-value*** | **p value^†^** | **p value^‡^** | **n** | **mean** | **SD** | **p-value*** | **p value^†^** | **p value^‡^** |
| Pro casp-3 | sham | 4 | 0.80 | 0.12 | 0.116 | - | - | 5 | 0.82 | 0.10 | 0.095 | - | - | 4 | 0.38 | 0.30 | **0.012** | - | - |
|  | moderate | 8 | 0.81 | 0.28 | **-** | **-** | **-** | 7 | 1.00 | 0.18 | **-** | **-** | **-** | 6 | 0.87 | 0.18 | **-** | **0.038** | 0.138 |
|  | severe | 7 | 1.08 | 0.21 | **-** | **-** | **-** | 7 | 1.03 | 0.19 | **-** | **-** | **-** | 7 | 1.08 | 0.25 | **-** | **0.024** |  |
| Cleaved casp-3 | sham | 5 | 0.61 | 0.17 | **0.001** | - | - | 5 | 0.86 | 0.26 | 0.655 | - | - | 5 | 0.72 | 0.10 | 0.162 | - | - |
|  | moderate | 8 | 0.97 | 0.21 | **-** | **0.004** | 0.152 | 8 | 1.21 | 0.61 | **-** | **-** | **-** | 6 | 0.84 | 0.46 | **-** | **-** | **-** |
|  | severe | 7 | 1.13 | 0.20 | **-** | **0.005** |  | 7 | 0.99 | 0.26 | **-** | **-** | **-** | 7 | 1.02 | 0.31 | **-** | **-** | **-** |

Western blot quantification of pro- and cleaved caspase-3 protein levels [ratio to β-actin]. Lung tissue samples were homogenized, separated, blotted and incubated. The number of observations (n), mean, and standard deviation (SD) are shown. Statistical analysis: Closed-testing principle among the trauma groups: *Kruskal-Wallis test (*p≤0.05), ^†^Wilcoxon-Mann-Whitney test (sham vs. moderate trauma, sham vs. severe trauma; ^†^p≤0.05), ^‡^Wilcoxon-Mann-Whitney test (moderate trauma vs. severe trauma; ^‡^p≤0.05).

S9 Table: Effects of mild hypothermia on lung tissue apoptosis

| **A)** |  | |  |  | | |  | |  | |  |  | | |  | |  | |  |  | | |  | |  | |  |
| --- | --- | --- | --- | --- | --- | --- | --- | --- | --- | --- | --- | --- | --- | --- | --- | --- | --- | --- | --- | --- | --- | --- | --- | --- | --- | --- | --- |
|  | |  | | |  | **Normothermia** | | | | | | |  | **Hypothermia** | | | | | | |  | **Difference** | | | | | |
| **Protein** | | **Trauma** | | |  | **n** | | **median** | | **SD** | | |  | **n** | | **median** | | **SD** | | |  | **mean** | | **95% CI** | | **p value** | |
| Pro casp-3 | | moderate | | |  | 8 | | 0.85 | | 0.28 | | |  | 10 | | 1.14 | | 0.30 | | |  | 0.29 | | -0.0 to 0.6 | | 0.071 | |
|  | | severe | | |  | 7 | | 1.04 | | 0.21 | | |  | 7 | | 0.88 | | 0.31 | | |  | -0.16 | | -0.3 to 0.4 | | 0.535 | |
| Cleaved casp-3 | | moderate | | |  | 8 | | 0.90 | | 0.21 | | |  | 11 | | 1.04 | | 0.15 | | |  | 0.14 | | -0.2 to 0.2 | | 0.600 | |
|  | | severe | | |  | 7 | | 1.10 | | 0.20 | | |  | 6 | | 0.98 | | 0.21 | | |  | -0.12 | | -0.4 to 0.2 | | 0.101 | |

| **B)** |  |  |  |  |  |  |  |  |  |  |  |
| --- | --- | --- | --- | --- | --- | --- | --- | --- | --- | --- | --- |
| **Protein** | **Trauma** |  | **Hypothermia** |  | **Normothermia** |  | **Difference*** |  | **Difference^†^** |  | **Interaction term** |
| Pro casp-3 | trauma |  | 0.10 |  | 0.05 |  | 0.05 |  | 1.05 |  | 1.05 - 1.00 |
|  | sham |  | 0.07 |  | 0.07 |  | 0.00 |  | 1.00 |  |  |
| Cleaved casp-3 | trauma |  | 0.15 |  | 0.14 |  | 0.01 |  | 1.01 |  | 1.01 - 1.03 |
|  | sham |  | 0.07 |  | 0.04 |  | 0.03 |  | 1.03 |  |  |

Western blot of pro- and cleaved caspase-3 protein levels [ratio to β-actin]. Lung tissue samples were homogenized, separated, blotted and incubated. Table A) shows the number of observations (n), mean, and standard deviation (SD) for both trauma groups under normothermic and hypothermic conditions. Statistical analysis: Wilcoxon-Mann-Whitney test (p≤0.05). Table B) shows the interaction terms for the interaction of the combined trauma groups (without further statistical analysis). Logarithmic (*) and exponentiated (†) differences in variance are shown.

S1 Fig: Caspase-3 Western Blot


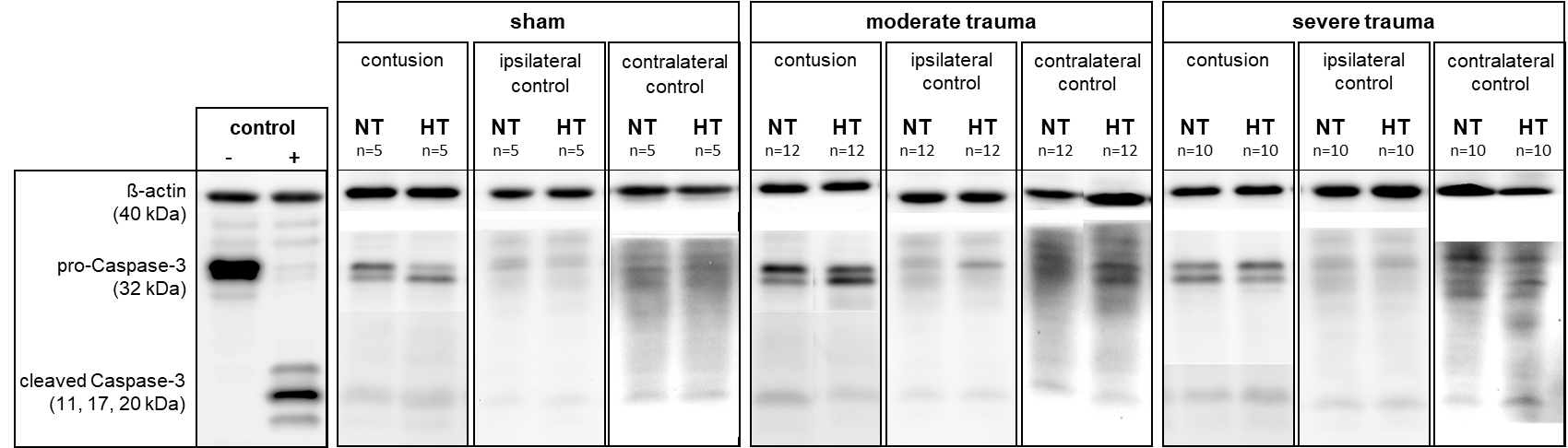


Western blot of pro- and cleaved caspase-3 [ratio to ß-actin]. Shown are representative blots separated by location of lung tissue sampling (contusion site, ipsilateral control, and contralateral control). Lung tissue samples were homogenized, separated, blotted and membranes were cut horizontally along 38 kDa to enable simultaneous analysis of ß-actin (40 kDa), pro-caspase-3 (32 kDa) and cleaved caspase-3 subunits (11, 17, 20 kDa). Untreated and cytochrome c-treated Jurkat control cell extracts served as controls. Statistical analysis based on a quantification of pro- and cleaved caspase-3 protein levels (ratio to ß-actin) applying a Wilcoxon matched-pairs signed rank test is shown in supplemental table 9 and supplemental figure 2.

S2-Fig: Apoptosis in lung tissue
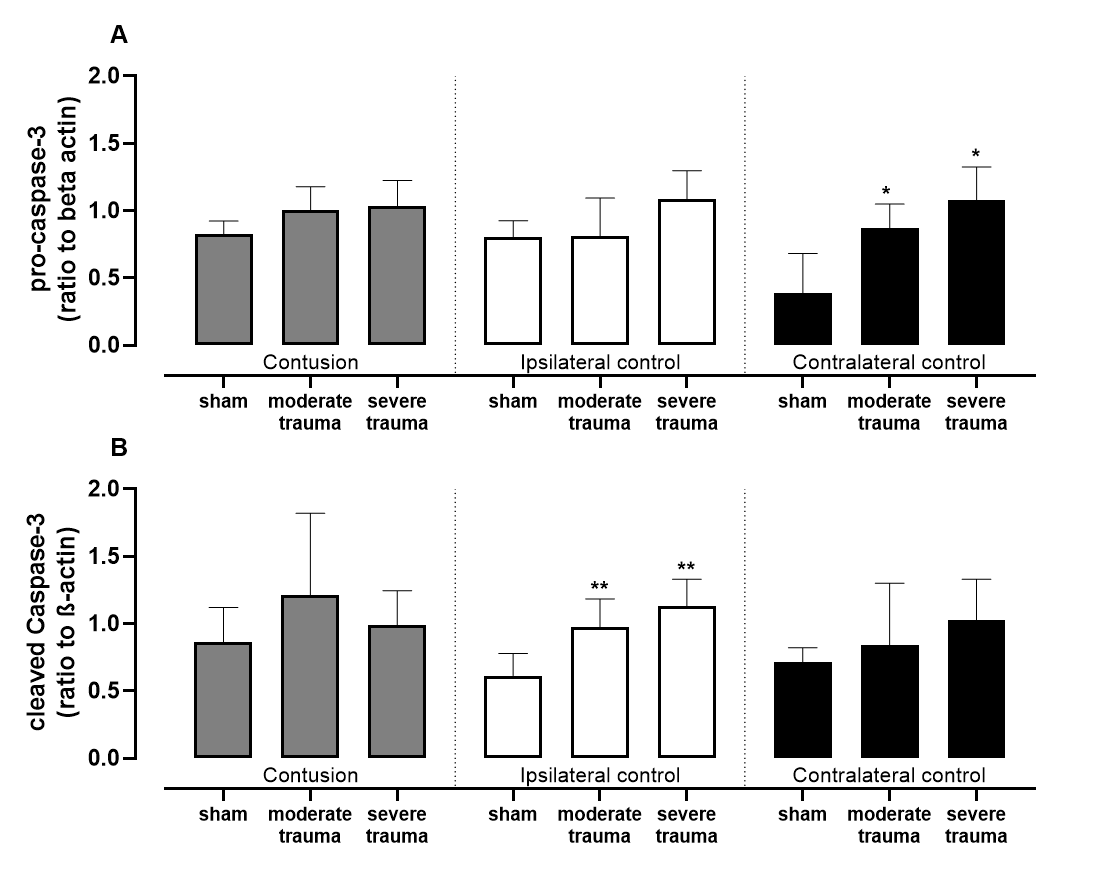


Western blot quantification of pro- and cleaved caspase-3 protein levels [ratio to ß-actin]. Lung tissue samples from the contusion site (right lower lobe), the ipsilateral control (right upper lobe) and the contralateral control (left upper lobe) were homogenized, and membranes were separated, blotted, incubated and quantified by densitometry. Mean values+SD on linear scale are shown. Statistical analysis: Wilcoxon matched-pairs signed rank test: *p<0.05, **p<0.01.

**References**

1. Bauer P. 1991. Multiple testing in clinical trials. Stat Med. 10(6):871-889; 889-890.
